# No Loan Comes Without a Price: Financial Literacy, Mental Health, and Consumer Loan Use Among Young Adults in Sweden

**DOI:** 10.3390/bs16030318

**Published:** 2026-02-26

**Authors:** Henrik Levinsson, Emma Samuelsson, Antonia Bjarup Nehlin, Signe Pröjts Erlandsson

**Affiliations:** Department of Psychology, Lund University, Box 217, SE-221 00 Lund, Sweden; emma.samuelsson@psy.lu.se (E.S.); antonianehlin@gmail.com (A.B.N.); signeprojtserlandsson@gmail.com (S.P.E.)

**Keywords:** consumer loans, financial literacy, mental health, loneliness

## Abstract

In Sweden, the number of young people taking consumer loans is growing. The reasons behind this increase remain unclear and may be explained by both financial and psychological factors. The purpose of the study was therefore to examine to what extent financial literacy, social and emotional loneliness and mental health were associated with the likelihood of having debt from consumer loans, after controlling for gender, educational level, and age. The study was based on an online survey comprising young adults (*n* = 2057) aged 18–29. Binary logistic regression analyses were conducted to identify the strongest associated factors of having debt from consumer loans. Lack of financial literacy and gender were the strongest associated factors. Men were twice as likely as women to have taken consumer loans. Anxiety and depressive symptoms, social and emotional loneliness, and age also emerged as significant associated factors. Educational level was not related to having debts from consumer loans. Overall, the results suggest that both financial and psychological aspects play a role in the likelihood of taking out consumer loans. Future research should investigate the causal pathways underlying these associations.

## 1. Introduction

In recent years, the macroeconomic situation in Sweden has been unstable. The debt levels have increased; from 2024 to 2025, the total debt held by Swedes at the Swedish Enforcement Authority increased by 19 billion SEK, and the number of individuals with registered debt rose by 20,000 during the same period (Swedish Enforcement Authority, 2025b). According to Klapper and Lusardi (2020), only one in three adults worldwide can correctly answer basic questions about interest rates, inflation, and risk diversification—regardless of whether they live in developing or economically advanced countries. This pattern is evident in the Swedish population as well (Samuelsson et al., 2024).

Sweden performs well in financial literacy on an international scale. However, young adults aged 18–29 exhibit lower financial literacy compared with those aged 30–59. This age-related gap is relatively large within Sweden, whereas it is smaller in many other countries (Swedish Financial Supervisory Authority, 2023c). A report from the Swedish Financial Supervisory Authority (2024a) claims that one in four Swedish adults has deficiencies in their knowledge of basic economic concepts. This lack of financial knowledge may contribute to the observed rise in consumer debt. Taking out consumer loans without full understanding of their terms can contribute to over-indebtedness, which may be regarded as a public health concern. Experiencing over-indebtedness early in life can have negative effects on the individual. In a study by Eriksson and Davidsson (2025), the authors found that over-indebted young adults experience a hard time emerging into adulthood, partly because of the financial strain’s effects on social relationships.

Research has shown that indebtedness is strongly associated with mental health problems, including anxiety, depression, and suicidal behavior, and that financial difficulties are a key predictor of persistent loneliness (Drentea & Reynolds, 2012; Levinsson et al., 2023; Lim et al., 2023; Rojas, 2022). The intersection between economics and psychology is thus crucial, as financial vulnerability can have extensive consequences for mental health and vice versa. Young adults appear particularly vulnerable, as this life stage is often marked by financial instability, identity formation, and increased psychological susceptibility (Swedish Financial Supervisory Authority, 2023a; Swedish Agency for Youth and Civil Society, 2022). Exploring the relationship between indebtedness, loan-taking, and mental health among young adults could provide valuable insights into the link between personal finances and psychological well-being, as well as ways to prevent over-indebtedness early in life.

This exploratory study addresses a clear research gap and examines how financial literacy, mental health, and loneliness are associated with consumer debt among young adults in Sweden, after controlling for gender, educational level, and age. Previous research has not sufficiently investigated how these factors influence consumer behavior in young adults, highlighting the need for further investigation into their financial and psychological well-being. The background section outlines and defines the key concepts and variables included in the study. By exploring how financial and psychological factors relate to consumer debt, the study aims to improve understanding of financial behavior and well-being.

### 1.1. Financial Literacy

Financial literacy refers to an individual’s ability to understand and manage their economic resources effectively. This encompasses making informed decisions about consumption, saving, and investing (Lusardi, 2019). The concept of financial literacy describes how economic knowledge, cognitive processes, and behaviors influence an individual’s financial decision-making. A key aspect of the concept of financial literacy is that it encompasses not only financial knowledge but also the ability to apply that knowledge through financial behaviors such as self-control, long-term planning, and rational decision-making (Hwang & Park, 2023; Lusardi, 2019). According to the Organisation for Economic Co-operation and Development (OECD, 2023), financial literacy is an essential component of financial well-being. Despite financial education becoming a policy priority in many countries, recent studies indicate that the growing complexity of financial systems has outpaced improvements in financial literacy (Lusardi & Mitchell, 2023). As previously mentioned, the situation in Sweden is concerning, with 25 percent of adults lacking knowledge of basic economic concepts.

Since the turn of the millennium, the subject of financial literacy has been extensively studied. A systematic review by Goyal and Kumar (2021) evaluated the field and found that the most extensively researched themes include levels of financial literacy across different demographic groups, the influence of financial literacy on financial planning and financial behavior, and the impact of financial education.

A meta-analysis by Santini et al. (2019), comprising 44 eligible studies, examined factors that function as antecedents and consequences of financial literacy. Educational attainment, financial knowledge, financial behavior, and household income emerged as significant antecedents. Higher levels of financial literacy were negatively associated with the incurrence of avoidable credit and checking account fees, positively associated with credit scores, and positively related to individuals’ willingness to take investment-related risks. However, no significant associations were found between financial literacy and credit card behaviors or financial well-being. This is contrasted by results from Hwang and Park (2023) where both objective and subjective financial literacy were positively related to financial well-being.

Lusardi (2019) demonstrated that low financial literacy can lead to adverse outcomes, including over-indebtedness and an inability to manage financial crises. At the same time, a study by Lo Prete (2013) indicates that by investing in financial education there may be long-term effects, such as reducing poverty and income inequality. By strengthening financial literacy in the population, more individuals can participate in financial markets, make more informed financial decisions, and contribute to economic growth and stability (Lo Prete, 2013; Lusardi, 2019).

Several studies have demonstrated gender differences in financial literacy and how these differences influence financial behavior (Almenberg & Dreber, 2015; Fonseca et al., 2012; Fornero, 2021; Grohmann, 2016; Lusardi et al., 2010; Lusardi & Mitchell, 2017). Women are generally more risk-averse and have lower confidence in their financial literacy compared to men (Bucher-Koenen et al., 2021; Swedish Financial Supervisory Authority, 2023a, 2023b; Tang & Baker, 2016). Lien Oskarsson (2023) found that this reluctance influences financial behavior, notably contributing to women’s underrepresentation in the stock market. Women also tend to have a greater tendency towards uncertainty or lack of confidence in financial knowledge (Bucher-Koenen et al., 2021; Mahdavi & Horton, 2014; Samuelsson et al., 2024). According to a study by Bucher-Koenen et al. (2021), one-third of the gender gap in financial literacy could be attributed to women’s lower financial self-confidence.

Another demographic variable that has proven important in regard to financial literacy is educational level. Kaiser and Lusardi (2024) demonstrated that a higher level of education is generally associated with better financial literacy. Individuals with higher education are more likely to make long-term investments and participate in the financial market. In contrast, Mahdavi and Horton (2014) studied financial literacy among highly educated women and found that despite holding at least a bachelor’s degree, only ten percent of the women correctly answered all four questions. One notable observation made by the authors was that more than half of the participants chose the response option “I don’t know” for all four questions (Mahdavi & Horton, 2014).

### 1.2. Consumer Loans

A report by the Swedish Financial Supervisory Authority (2020) indicates that personal loans and credit-based borrowing are on the rise, with increased access to digital lending being a possible contributing factor. The report also highlights age-related differences in consumer lending: younger individuals tend to take out smaller loans, whereas older adults are more likely to borrow for housing or debt consolidation. Rising consumer debt poses risks for both individual households and lenders.

Consumer loans are credits obtained to finance personal consumption, such as purchase of goods and services. Consumer loans come in different types, which Andersson and Üye (2021) divide into two main groups: unsecured loans and loans secured by collateral. Unsecured loans include invoices, installment plans, credit lines, and personal loans without any collateral. Secured loans, often referred to as object loans, are substantial consumer loans that are collateralized by valuable assets, such as vehicles or real estate. Repayment terms for these loans can vary widely, from paying back in less than a month to spreading payments over several months or even years (Statistics Sweden, 2022).

A large portion of the increased debt in Sweden comprises consumer loans (Swedish Enforcement Authority, 2025b), and there is limited understanding of the underlying reasons for this. One possible explanation is provided by the Swedish Financial Supervisory Authority (2024b, 2024c), which argues that low financial literacy can lead to individuals making poorly considered financial decisions. Psychological factors also appear to be linked to borrowing. For instance, the Public Health Agency of Sweden (2024) reports a correlation between loneliness and financial vulnerability.

Lack of financial literacy is linked to loan behavior. Salas (2024) found that young adults who received financial education were less likely to take on excessive student loans. Similarly, Disney and Gathergood (2012) found that individuals who used consumer credit had significantly lower financial literacy than those who did not. Martin et al. (2021) found that individuals lacking financial literacy were more likely to take out high-interest loans compared to those with financial literacy.

There are notable gender differences in the propensity to take consumer loans. Two thirds of individuals with debts registered with the Swedish Enforcement Authority are men (Swedish Enforcement Authority, 2025a). At the same time, the total amount of debt among women is increasing at a much faster rate than among men. Furthermore, the proportion of women with debts is growing more rapidly than the proportion of indebted men (Swedish Enforcement Authority, 2022). Although statistics from the Enforcement Authority do not differentiate between types of loans, previous research has demonstrated that gender differences are particularly visible when it comes to consumer loans (Andersson & Üye, 2021). The size of the loan amount and the frequency of taking loans differ between men and women: men take out larger consumer loans, whereas women take loans more frequently. One reason for this difference can be found in the purpose of the loans. It is more common for women to lend money when doing online purchases, often in the form of installment payments or invoicing, whereas men are more likely to borrow from major banks (Andersson & Üye, 2021).

In this study, consumer loan debt refers to unsecured loans used for consumption. Debt from consumer loans is typically associated with higher borrowing costs, limited collateral and increased vulnerability to financial strain. However, debt is not inherently indicative of financial distress. In some contexts, debt can reflect financial planning or investment and be considered beneficial when it is used to acquire assets that appreciate in value, generate income or improve long-term financial stability, such as mortgages for homes, student loans or investment in business (Francis-Devine, 2025). In this study, debt from consumer loans is associated with short-term, high-cost credit that does not generate long term value, and could therefore contribute to financial difficulties, due to higher interest rates and shorter repayment terms (Disney & Gathergood, 2012; OECD, 2023).

### 1.3. Anxiety and Depression Among Young Adults

Many young adults in Sweden lack the knowledge, opportunities, and abilities required to manage their financial situation effectively, which can lead to persistent worry and stress about not having enough money (Swedish Agency for Youth and Civil Society, 2022). Lusardi and Mitchell (2014) found a link between financial stress and mental health problems. Financial worries can contribute to anxiety, depression, and an increased risk of social isolation (Lusardi & Mitchell, 2014). The link between mental illness, both as a self-perceived experience and as a diagnosis, and poor financial conditions, debt, and low socioeconomic status is well-researched (Cesar Leandro & Botelho, 2022; Drentea, 2000; Holmgren et al., 2019; Holzer et al., 1986; Jenkins et al., 2008; Ljungqvist et al., 2016; Mills, 2015; Östergren et al., 2022).

However, the causality of this relationship is not fully established, although published research findings suggest a bidirectional influence, where both factors reinforce each other, potentially resulting in a vicious cycle over time (Bialowolski et al., 2025) As mentioned above, lacking sufficient financial knowledge was, in a Swedish context, shown to lead to stress and worry regarding one’s financial situation. The link between financial knowledge and mental health has, for example, also been shown in the United States, where a study found subjective financial knowledge, i.e., confidence in having adequate financial knowledge, to be impactful on life satisfaction, a measure which reliably indicates mental well-being (Heo et al., 2025).

In 2020, the proportion of young people in Sweden with a low economic standard increased, and currently, this affects more than one in four young adults between the ages of 20 and 24 (Swedish Agency for Youth and Civil Society, 2022). The reasons many young people experience poor mental health include, among other things, unemployment, difficulties supporting themselves, and over-indebtedness (Hagquist & Gustafsson, 2020; Paananen et al., 2013).

### 1.4. Loneliness

One in three young adults aged 16–29 in Sweden experiences loneliness and social isolation. According to the report, young men and women appear to be equally affected (Public Health Agency of Sweden, 2024), and several studies have similarly shown a lack of gender differences in relation to loneliness (Anyan & Hjemdal, 2022; Nazzal et al., 2021). That young adults are a particularly affected demographic group when it comes to loneliness is also supported by the findings of a study by Luhmann and Hawkley (2016), which found that young adults experience a higher degree of loneliness than other age groups.

Qualitative studies on the topic can provide valuable insight into how young adults experience loneliness. In a study by Käcko et al. (2024), the experiences of involuntary loneliness among young adults in Finland were explored. The study showed that these experiences are often characterized by feelings of alienation and of being different or invisible. Similar findings were presented by Fardghassemi and Joffe (2021), who found that young adults’ experiences of loneliness were permeated by negative emotions, thoughts, and perceived isolation. There thus appears to be support for the idea that loneliness is linked to mental illness.

Loneliness is not a one-dimensional experience; it can take different forms. Two commonly discussed constructs are emotional loneliness and social loneliness (Gierveld & Van Tilburg, 2006). Emotional loneliness refers to the perceived absence of close, emotionally supportive relationships. This type of loneliness is often experienced when someone lacks a deep connection with another person. In contrast, social loneliness is related to the perceived lack of a broader social network. It involves feelings of isolation due to the absence of a network of friends or acquaintances. Importantly, these two forms of loneliness do not always overlap. A person may have an active social life and a wide network of acquaintances but still feel emotionally lonely if they lack meaningful, intimate relationships (Gierveld & Van Tilburg, 2006; Green et al., 2001; Weiss, 1973).

In a cohort of young adults, Fan and Ryu (2023) investigated how financial debts, specifically student loans, impact subjective well-being across psychological, emotional, and social domains. They discovered that while student loans increased the likelihood of receiving financial support and enhanced self-esteem, financial support was negatively associated with life satisfaction and overall subjective well-being. An Australian report by Lim et al. (2023) found that financial difficulties were the strongest predictor of persistent loneliness and social isolation; individuals experiencing financial difficulties were nearly seven times more likely to report long-term loneliness, even after controlling for confounding variables.

However, economic consumption can also act as a coping mechanism for loneliness. Shrum et al. (2022) found that individuals experiencing loneliness often engage in compensatory consumption, including ritualistic, avoidance, impulsive, and uniqueness-driven purchases, to fulfill unmet social connection needs. While such spending can provide short-term relief, it carries substantial psychological costs, including heightened mistrust and depleted self-regulatory capacity.

### 1.5. Aims and Research Questions

Lack of financial literacy increases the risk of financial difficulties, especially as consumer loans have become easier to access but harder to understand. Many young adults lack basic financial skills, and low financial literacy can lead to adverse outcomes, including over-indebtedness (Lusardi, 2019), which in turn has been linked to mental ill-health (Drentea, 2000; Östergren et al., 2022). This exploratory study investigates how financial literacy, mental health, and loneliness are associated with consumer loan behavior among Swedish young adults. To the best of the authors’ knowledge, these associations have not been previously studied in a Swedish context, and findings may also have relevance for international research.

The results aim to inform strategies to prevent financial and psychological difficulties and to raise professional awareness of these challenges. Based on these considerations, the following research questions were formulated:

1. Are financial literacy, anxiety and depressive symptoms, emotional and social loneliness, gender, educational level, and age associated with consumer loan debt?
2. Which of financial literacy, anxiety and depressive symptoms, and emotional and social loneliness are most strongly associated with having debt from consumer loans, after controlling for gender, educational level, and age?

## 2. Method

### 2.1. Design

The study was designed as a quantitative cross-sectional study. The quantitative method was applied based on the study’s aim of finding relationships and associated factors of the included variables based on a larger sample size. All data were collected during two weeks in October 2021 via a web survey. The web survey was distributed by the external research company Enkätfabriken, which specializes in collecting data through web panels and consists of individuals who have agreed to participate in surveys (Enkätfabriken, n.d.). The survey was created using the Qualtrics platform and included 117 questions. Before completing the survey, participants were informed about its content, the purpose of the study, and that participation was anonymous, voluntary, and could be withdrawn at any time. They received a small compensation from the panel provider, typically in the form of points redeemable for gift cards or donations to NGOs, usually around 0.5–1 SEK per minute of participation, which is standard practice for most web panels. The median completion time for the survey was approximately 11 min. Some participants completed it over multiple sessions, making the mean completion time less informative.

The survey consisted of questions measuring financial literacy, personal financial situation, loneliness and mental health. Participants also provided information on demographic variables such as age, gender, educational level, marital status, and employment status.

### 2.2. Participants

The participants were recruited from Enkätfabriken’s web panel, which consisted of 200,000 individuals. The sample included 2057 young adults aged 18–29. In total, 1059 women and 998 men participated, with a mean age of 24 years. To ensure the sample was as representative as possible, Enkätfabriken controlled for demographic variables such as geographic location, age, and gender. See Table 1 for demographic data on the participants.

### 2.3. Instruments

#### 2.3.1. Financial Literacy

Financial literacy was measured using the “big three” by Lusardi and Mitchell (2008, 2011). The scale consists of three questions concerning interest rate, inflation, and risk diversification. The first question was adapted to Swedish currency (Swedish kronor, SEK) to suit the national context. The scale has been used in several studies and reports and is described as having good reliability and validity for measuring financial literacy (Kaiser et al., 2025; Lusardi & Mitchell, 2014). The three questions are:

1. Suppose you had 100 Swedish kronor in a savings account and the interest rate was 2% per year. After 5 years, how much do you think you would have in the account if you left the money to grow?
  (a) More than 102 Swedish kronor (correct answer)
  (b) Exactly 102 Swedish kronor
  (c) Less than 102 Swedish kronor
  (d) Don’t know
2. Imagine that the interest rate on your savings account was 1% per year and inflation was 2% per year. After 1 year, how much would you be able to buy with the money in this account?
  (a) More than today
  (b) Exactly the same
  (c) Less than today (correct answer)
  (d) Don’t know
3. Is the following statement true or false? “Buying a single company’s stock usually provides a safer return than a stock mutual fund.”
  (a) True
  (b) False (correct answer)
  (c) Do not know

#### 2.3.2. Consumer Loans

Respondents’ consumer loan status was assessed with a yes/no question about loans taken more than 18 months ago. This aimed to target respondents with long-term debt, as this could be indicative of financial difficulties.

Although the term consumption loan is used throughout this manuscript, the Swedish term refers specifically to high-cost, short-term loans with easy access and high interest rates, commonly described as payday loans, rather than to consumer credit more broadly.

#### 2.3.3. De Jong Gierveld Loneliness Scale (DJGLS)

Loneliness was measured with the De Jong Gierveld Loneliness Scale (DJGLS), developed by De Jong Gierveld and Van Tilburg (1999). The scale is a self-report measure assessing perceived loneliness. It does not take into account a person’s actual social situation, such as the number of friends or frequency of social contact. The scale can be used as a full scale or divided into two subscales, where six items measure emotional loneliness (e.g., “I miss having a really close friend”), and five items measure social loneliness (e.g., “There is always someone I can talk to about my day-to-day problems”). The scale uses the same five response options for all eleven items: “yes!”, “yes”, “more or less”, “no”, and “no!”. The two subscales were used separately since the present study aimed to explore different facets of loneliness in greater detail. The reliability of emotional loneliness in the present study was α = 0.83, and social loneliness was α = 0.82. The reliability of the entire scale was α = 0.82. Other studies investigating the DJGLS have found similar evidence supporting good reliability for the instrument (Giraldo-Rodríguez et al., 2023; Uysal-Bozkir et al., 2017).

#### 2.3.4. The Hospital Anxiety and Depression Scale (HADS)

To measure anxiety and depressive symptoms, The Hospital Anxiety and Depression Scale (HADS) was used. It is divided into two subscales: Anxiety (HADS-A) and Depression (HADS-D), each comprising seven items. Scores for the entire HADS are not combined; instead, each subscale produces a score ranging from 0 to 21. Participants respond based on how they have felt over the past week, using a 4-point Likert scale from 0 to 3. Scores between 0 and 7 suggest that clinical anxiety or depression is unlikely, scores from 8 to 10 indicate a possible presence, and scores of 11 or higher suggest that clinical anxiety or depression is likely. These cutoff values have been applied in both Swedish and international research (Bodlund, 1997; Lisspers et al., 1997). It is important to note, however, that there is no clear dividing line between individuals with and without anxiety or depression (Zigmond & Snaith, 1983). In the present study, HADS was treated as a continuous variable.

HADS has demonstrated strong internal consistency, with Cronbach’s alpha values ranging from 0.80 to 0.93, and correlates well with other measures such as the Beck Depression Inventory (BDI) and the General Health Questionnaire (GHQ) (Bjelland et al., 2002; Herrmann, 1997; Lisspers et al., 1997). In this study, the internal reliability was α = 0.79 for the anxiety subscale and α = 0.75 for the depression subscale.

The variable educational level was treated as an ordinal variable with three categories: compulsory school, upper secondary school, and higher education. The variable gender consisted of two categories: male and female. Age was treated as a continuous variable.

### 2.4. Data Analysis

Correlation and logistic regression were used to answer the research questions. Data was analyzed using the statistical software IBM SPSS 29. For the variable financial literacy, analyses were conducted using each of the three questions as separate factors, as well as using the scale as a composite sum variable. The classification proposed by Lusardi was also assessed (Lusardi & Mitchell, 2011). Lusardi’s dichotomous categorization consisted of two groups: those who answered all three questions correctly were placed in the group “financially literate,” while those who answered at least one question incorrectly or responded “I don’t know” were placed in the group “not financially literate.” The analyses comparing the sum scale and Lusardi’s dichotomous scale yielded very similar results. Therefore, it was not considered psychometrically problematic to divide this scale into two dichotomous groups. Moreover, this categorization is aligned with the notion that “the big three” are to be regarded as such basic questions that an incorrect or “I don’t know” response indicates a lack of financial literacy. Based on this reasoning, the dichotomous categorization was subsequently used in the statistical analyses.

For the DJGLS variable, particular attention was given to the two subscales measuring emotional and social loneliness, in order to explore distinct aspects of loneliness separately. Emotional and social loneliness share limited variance and thus appear to reflect distinct aspects of loneliness (Wolters et al., 2023). In the present study, the correlation between the two subscales was *r* = 0.42, which is consistent with previous findings by Dahlberg and McKee (2014) and Green et al. (2001), who reported similar correlations (*r* = 0.44 and *r* = 0.41, respectively).

To answer the first research question to explore potential relationships between associated factors and the outcome variable, correlations were analyzed using Pearson’s correlation coefficient, *r*. For associations between two dichotomous variables, *r* corresponds to the phi coefficient. For the ordinal variable educational level, Kendall’s tau-b was used, as it is appropriate for measuring associations involving ordinal variables. The following cutoff values from Cohen (1988) were used to interpret the strength of the correlations: small (*r* = 0.10 to 0.29), medium (*r* = 0.30 to 0.49), and large (*r* = 0.50 to 1.00).

Before conducting analyses, assumptions for correlation analysis were checked. Normal distribution was assessed for the continuous variables emotional and social loneliness, and anxiety and depressive symptoms. By inspecting histograms and examining skewness and kurtosis, the data were deemed to be normally distributed. Linear relationships between the continuous variables were also examined.

To answer the second research question, hierarchical logistic regression analysis was applied in order to examine the associations of financial literacy, anxiety and depressive symptoms, and emotional and social loneliness with the outcome variable of having consumer loans, after controlling for gender, educational level and age. Consumer debt status was coded as 0 = no debt and 1 = debt, and financial literacy was coded as 0 = have financial literacy and 1 = lack financial literacy. Female was used as the reference category for the gender variable.

Variables were entered in four blocks to evaluate their contribution to the model, using the enter method. In Block 1, the control variables (gender, education, and age) were entered to account for demographic influences. In Block 2, financial literacy was added, followed by anxiety and depressive symptoms in Block 3, and finally, emotional and social loneliness in Block 4. Several models incorporating different combinations of explanatory variables were estimated. Model specification was guided by theoretical considerations and statistical significance, with the aim of ensuring transparency and minimizing the risk of overfitting. This approach enabled the identification of the most relevant factors based on prior research, while avoiding purely data-driven or exploratory model selection.

The assumptions for logistic regression were checked. The sample consisted of 2057 individuals. This large sample size suggested that outliers would not be a problem. To further ensure outliers would not influence the analysis, residuals were checked through a multiple regression analysis, and no signs of outliers were found. Variance inflation factors (VIFs) were used to assess multicollinearity to ensure it did not reach levels that would compromise the admissibility of the estimations. The highest VIF observed was 1.883 (depression symptoms), well below common thresholds (VIF > 5) indicating that multicollinearity was not a concern in the model (Kim, 2019; O’Brien, 2007). Odds ratios with 95% confidence intervals (ORs) were used to assess the strength of associations between the independent variables and the outcome. Nagelkerke’s *R*^2^ was employed to evaluate the proportion of pseudo-explained variance in the models.

### 2.5. Ethical Considerations

The study was not subject to ethical review under Swedish law (2003:460) (Swedish Parliament, n.d.), as the survey was anonymous and no personal data were collected. Participants were informed that their participation was voluntary and that they could withdraw at any time without providing a reason. The survey included questions on topics that could, in some cases, lead to distress, such as personal finances, loneliness, depression, and anxiety. Therefore, at the beginning of the survey, participants were informed that if they experienced distress, they could seek help from healthcare services. After reading this introductory information, participants gave informed consent before proceeding with the actual survey.

## 3. Results

### 3.1. Descriptive Statistics

Descriptive data for the sample is presented in Table 2 and Table 3. Table 2 shows the proportion of participants with and without consumer loans. Table 3 provides information about the various associated factors. One fifth of participants answered all three questions concerning financial literacy correctly and were considered to have financial literacy. The majority of the sample responded incorrectly or “I don’t know” to at least one of the questions and were therefore considered to lack financial literacy.

#### 3.1.1. Correlations Between Consumer Loan Debt and Financial, Psychological and Demographic Variables

The correlations are presented in Table 4. Significant correlations were found between financial and psychological variables and having debts from consumer loans, except for social loneliness, which was close to the threshold for statistical significance. In addition, educational level and age were weakly associated with having debt from consumer loans. Chi^2^ tests were conducted to examine the associations between gender and both financial literacy and having debt from consumer loans. The results indicated significant gender associations in both outcomes. Men were more likely than women to report having taken out consumer loans (see Table 5).

#### 3.1.2. Associations of Financial Literacy, Anxiety and Depression Symptoms, and Emotional and Social Loneliness with Consumer Loan Debt, Controlling for Gender, Education, and Age

A hierarchical logistic regression analysis was conducted to determine whether financial literacy, anxiety and depressive symptoms, and emotional and social loneliness were associated with the likelihood of having taken consumer loans, after controlling for gender, educational level, and age. The final model (Block 4) is presented in Table 6. At each step, the model fit improved, as indicated by a decrease in the Akaike Information Criterion (AIC), suggesting that the included variables contributed additional explanatory value to the probability of having consumer loans. Supplemental Materials show AIC and Nagelkerke *R*^2^ for all blocks.

The model was statistically significant, χ^2^(8, N = 2057) = 311.93, *p* < 0.001, indicating that the set of independent variables was significantly associated with the likelihood of having debt from consumer loans. The model explained 21% of the variance (Nagelkerke *R*^2^ = 0.21).

Lacking financial literacy and gender were the strongest associations with having consumer loan debt, with individuals lacking financial literacy having 149% higher odds (OR = 2.49, 95% CI [1.78, 3.49], *p* < 0.001) and males nearly doubling the odds (OR = 1.98, 95% CI [1.58, 2.46], *p* < 0.001). Anxiety and depression symptoms and emotional loneliness increased the odds by 8–21%, while social loneliness decreased the odds by 23%. Age was also significant, whereas education was not.

## 4. Discussion

Descriptive statistics for the sample indicate that only approximately 20% of respondents possess financial literacy, which contrasts with global estimates reported by Klapper and Lusardi (2020), suggesting that roughly one in three individuals is financially literate. However, prior research (Swedish Financial Supervisory Authority, 2023c) indicates a relatively large age gap where young adults in Sweden tend to exhibit lower levels of financial literacy than the general population. This observation is further supported by statistics from the Swedish Financial Supervisory Authority (2024a), which report that one in four Swedish adults demonstrates deficiencies in their knowledge of basic economic concepts.

The results show that lacking financial literacy is significantly associated with having debt from consumer loans. According to the odds ratio values, this variable appears to have the strongest association with the outcome variables. The notion that financial literacy may be related to loan behavior is consistent with previous research showing that lower financial literacy is associated with the use of consumer credit and a higher likelihood of taking out high-interest loans (Disney & Gathergood, 2012; Martin et al., 2021). Although this supports the idea of financial literacy influencing loan taking, it is possible that a reverse causality could be present. Holding long-term debt could, in some ways, enhance financial literacy. For some people, taking loans could serve as a motivator to properly research the loan market and understand terms and conditions of loan taking such as interest rates. However, support for a reverse causality such as that has not been found in the existing research literature during the course of this study.

Next to financial literacy, gender was found to be one of the most significant associated factors in the regression models, with men being twice as likely as women to report having debt from consumer loans. This finding is consistent with national data indicating that two thirds of individuals with debts registered at the Swedish Enforcement Authority are men (Swedish Enforcement Authority, 2025a). At the same time, other statistics highlight a growing trend of indebtedness among women, who more frequently use consumer credit, albeit in smaller amounts (Andersson & Üye, 2021; Swedish Enforcement Authority, 2022). These findings can be interpreted in light of prior research showing robust gender differences in financial literacy, with men consistently performing better than women on financial literacy measures (Klapper & Lusardi, 2020; Lusardi & Mitchell, 2023). However, despite being more financially literate, men were more likely to take out consumer loans. Other factors, such as overconfidence or social norms around risk-taking, may influence the propensity of taking loans (Tahir, 2025; Hansen, 2025).

Although earlier research (Lim et al., 2023; Loibl et al., 2021) has demonstrated a positive relationship between financial difficulties and loneliness, there has been more data supporting the relationship of subjective financial strain rather than objective measurements such as the actual size of the debt. Yet, the findings of Lim et al. that financial difficulties were a strong predictor of loneliness support some of the results of the present study.

The present study separated the two DJGLS-subscales and the results show that social and emotional loneliness have different and even opposite associations with having debt from consumer loans. The positive association between emotional loneliness and consumer loans may suggest that individuals who lack such close relationships might use consumer loans as a way to cope with feelings of emptiness. Loibl et al. (2021) found that social loneliness was related to subjective but not to objective debt burden, which is not consistent with the findings from the present study. This could be explained by the two studies using different aspects to measure objective debt burden.

Emotional loneliness can contribute to emotional vulnerability, where borrowing and consumption become means of seeking comfort or temporary satisfaction, so-called “retail therapy”. The reported over-use of compensatory consumption by Shrum et al. (2022) of individuals experiencing loneliness supports this theory although the researchers did not differentiate between/separate emotional from social loneliness.

It is possible that people experiencing social loneliness have fewer social opportunities and therefore less motivation to take consumer loans. Alternatively, socially isolated individuals may have a more restrictive consumption pattern due to practical or financial reasons. Overall, these findings highlight the importance of distinguishing between different aspects of loneliness in studies of financial behavior.

Anxiety and depressive symptoms were also significantly associated with consumer loan behavior, aligning with previous research demonstrating a strong association between mental health challenges and financial difficulties. Prior studies have indicated that many young adults lack the knowledge, resources, and opportunities required to manage their financial situation, which may result in persistent financial stress and psychological strain (Swedish Agency for Youth and Civil Society, 2022). Lusardi and Mitchell (2014) identified financial stress as a contributing factor to anxiety, depression, and social isolation, findings that are consistent with the present results. Furthermore, Drentea and Reynolds (2012) found debt to be the most influential factor related to increased symptoms of anxiety and depression, suggesting that financial strain can lead to internalized emotional responses. The current findings support the notion that mental health problems are not only associated with general financial difficulties but also specifically linked to consumer loans, indicating a potentially reinforcing cycle between psychological vulnerability and debt accumulation. However, because of the cross-sectional nature of the present study, reverse causality regarding the links between anxiety and consumer loan taking has to be considered when interpreting these results. It is impossible to, from the present study, derive what came first: the symptoms of anxiety and depression, or consumer loan indebtedness.

In the present study, the relationship between educational level and taking consumer loans was not significant. However, educational level has previously been linked to consumer loans in a report by Almenberg et al. (2016), which found that consumer loans were most common among individuals with only an upper secondary education. The Government Inquiry on Over-Indebtedness (Utredningen om överskuldsättning, 2013) also found that this group was overrepresented among those with large consumer loans, whereas individuals with post-secondary education were underrepresented.

In the present study, financial literacy was weakly but significantly associated with educational level. Kaiser and Lusardi (2024) found that highly educated individuals tend to have greater financial literacy compared to those with lower educational levels. Lusardi and Mitchell (2023) reported that 65 percent of individuals with a university degree answered all questions correctly on “the big three” financial literacy measures, in contrast to only 18 percent of those who had not completed secondary education. However, the same study also found that more than one-third of the highly educated group answered at least one question incorrectly, suggesting that higher education alone does not guarantee financial literacy. Similar findings were reported by Mahdavi and Horton (2014) in a study of highly educated women, concluding that higher education does not necessarily lead to financial literacy. In summary, while educational level may influence the association between financial literacy and consumer loan debt, other factors are also likely to play a significant role.

### 4.1. Limitations

A potential limitation of this study is that the large sample size could increase the risk of detecting statistically significant associated factors. Supporting this concern is the fact that the effect sizes are small. This could indicate a Type I error, meaning that associated factors appear statistically significant even though they contribute very little explanatory value in reality. Despite the risk of Type I errors in large samples, having a smaller sample would have been a greater disadvantage from a statistical perspective, as it would have reduced statistical power. It might also have increased the risk of missing actual associations and limited the generalizability of the results.

A limitation of this study is also the use of a web panel for data collection. While this method allows for efficient sampling of a relatively large number of respondents, it may limit the generalizability of the findings to the broader population of young adults. Selection bias is also a concern, as participants who choose to engage in web panels may differ from those who do not, potentially affecting the representativeness of the sample. Caution is therefore warranted when extrapolating results beyond the sample studied. The variable targeting consumer loans is self-constructed and was limited to a span of 18 months (1.5 years) in an attempt to address persistent and prolonged financial issues. However, this timespan can be subject to further reflection. Additionally, as this is a cross-sectional study, no causal inferences can be drawn from the observed associations. Nevertheless, studying a larger sample provides valuable insights into the relationships between the variables and contributes important knowledge within the scope of the current research. Rather, the findings should be considered hypothesis-generating and serve as a basis for further research.

Finally, the data used were collected in October 2021, when the COVID-19 pandemic was ongoing. It is possible that participants’ likelihood of taking consumer loans and their experience of being part of a larger context were affected by the societal restrictions in place. As such, the results might have been different if more recent data had been used. On the other hand, the Public Health Agency of Sweden (2024) reports that perceived loneliness is common among young people. This underscores the continued relevance of investigating loneliness and financial circumstances in this population, even beyond the immediate context of the pandemic.

### 4.2. Future Research and Practical Implications

To better understand the mechanisms behind rising debt levels in Sweden, research using more recent data is needed. The total debt among young adults has increased, with a substantial portion consisting of consumer debt (Swedish Enforcement Authority, 2022). Studies examining these relationships in a contemporary context could provide valuable insights into the psychological factors associated with borrowing in today’s consumer society. Furthermore, in addition to cross-sectional research, experimental and longitudinal studies would be desirable to establish causal relationships and better capture the dynamic nature of financial behavior and mental health over time.

Previous studies have shown that women are less confident in their financial knowledge (Bucher-Koenen et al., 2021; Lusardi & Mitchell, 2023). It would be interesting to conduct further studies on the topic of self-confidence related to financial literacy. Asking participants to self-assess their uncertainty could also shed light on whether their responses are due to an actual lack of financial literacy or low confidence. This could also be explored qualitatively through focus groups to gain more insight into gender differences related to financial literacy and confidence.

Future studies could investigate additional variables to see if there are other variables associated with taking out consumer loans. Previous research suggests that lifestyle factors, norms, and attitudes toward borrowing and saving may play a role (Ackert et al., 2024; Bernthal et al., 2005; Kamleitner et al., 2012; Minibas-Poussard et al., 2018). A materialistic worldview may also be a factor. People with more materialistic values have been shown to have more positive attitudes toward borrowing (Kamleitner et al., 2012), which in turn has been linked to poor impulse control and a diminished ability to delay gratification (Watson, 2003).

Financial literacy education in schools has been studied by Lusardi (2015), who found it beneficial to help children develop financial skills early in life. An OECD report showed that countries that implemented financial education in schools demonstrated higher levels of financial literacy than those that did not (Lusardi, 2015). Brown et al. (2014) also found that mandatory financial education in schools was associated with fewer late loan payments among young people. Fornero and Lo Prete (2023) highlighted examples of how financial education for children can be introduced, such as through games, pocket money, personal bank accounts, and involving children in open family discussions about money. Early involvement in the family’s finances can improve financial literacy.

A practical implication of the findings is the need for greater attention to financial matters within healthcare (Levinsson et al., 2025). If healthcare professionals become more attentive to inquiring about and assessing patients’ financial situations, individuals experiencing mental health issues related to financial problems may be identified, thereby enabling the provision of timely and appropriate support.

### 4.3. Conclusions

The findings of this study highlight several important insights. Financial literacy is an important variable when it comes to having debt from loans, which implies that greater emphasis should be placed on improving financial literacy, both in Sweden and globally. The findings show that men are more likely than women to take consumer loans. This knowledge can help inform initiatives to prevent debt accumulation, which is important, as research has shown strong links between over-indebtedness and mental health problems. Targeted efforts by policymakers and public agencies are needed to reduce consumer borrowing among men. Earlier research has established a relationship between loneliness and both physical and mental health issues, and the results of this study further indicate an association between loneliness and financial well-being. Finally, financial literacy may represent a key protective factor and an essential piece of the puzzle in promoting long-term mental and financial well-being, as well as in preventing indebtedness early in life.

## Figures and Tables

**Table 1:** Demographic data on the participants (n = 2057).

Characteristics	n	%	M (SD)	Min–Max
Gender				
Female	1059	51.5		
Male	998	48.5		
Age			23.6 (3.5)	18–29
Completed education				
Compulsory education	255	12.4		
Further/upper secondary education	1032	50.2		
Higher education	770	37.4		
Marital status				
Married/partner	917	44.6		
Unmarried/single	986	47.9		
Other	154	7.5		
Type of housing				
Detached house/townhouse	429	20.9		
Condominium apartment	386	18.8		
Rental apartment	843	41.0		
Flatmate/sublet	73	3.5		
Living with parents	279	13.6		
Employment status				
Full-/part-time employee	959	46.6		
Self-employed	156	7.6		
Student	632	30.7		
Unemployed	202	9.8		
Source of income				
Salary	1121	54.5		
Financial assistance	215	10.5		
Student loans	440	21.4		

**Table 2:** Descriptive statistics on consumer loans.

Variable	Yes (n)	Yes (%)	No (n)	No (%)
Consumer debt	542	26.3	1515	73.7

**Table 3:** Descriptive statistics on financial literacy, loneliness, anxiety and depression symptoms, gender, and educational level.

Variable	n	%	M (SD)	Min–Max
Financial literacy				
Have financial literacy	410	19.9		
Do not have financial literacy	1647	80.1		
Total	2057	100		
Loneliness				
Emotional	2057	100	3.86 (2.00)	0–6
Social	2057	100	2.32 (1.76)	0–5
Anxiety	2057	100	10.29 (4.30)	0–21
Depression	2057	100	7.25 (3.93)	0–21
Gender				
Female	1059	51.5		
Male	998	48.5		
Total	2057	100		
Education				
Compulsory education	255	12.4		
Further/upper secondary education	1032	50.2		
Higher education	770	37.4		
Total	2057	100		

**Table 4:** Correlation matrix of financial, psychological and demographic variables and having debt from consumer loans.

Variable	1	2	3	4	5	6	7	8	9
1. Financial literacy	1.00								
2. DJGLS Emotional	0.16 ***	1.00							
3. DJGLS Social	0.02	0.42 ***	1.00						
4. Depression	0.21 ***	0.50 ***	0.40 ***	1.00					
5. Anxiety	0.16 ***	0.51 ***	0.32 ***	0.60 ***	1.00				
6. Consumer loans	0.16 ***	0.21 ***	−0.04	0.23 ***	0.22 ***	1.00			
7. Gender	0.11 ***	0.01	−0.06 *	0.06 **	−0.10 ***	0.12 ***	1.00		
8. Education	−0.12 ***	−0.02	−0.06 **	−0.05 **	−0.06 ***	−0.02	0.04	1.00	
9. Age	−0.11 ***	−0.03	0.02	−0.08 ***	−0.06 **	0.05 *	−0.04	0.20 ***	1.00

Note: * *p* ≤ 0.05, ** *p* ≤ 0.01, *** *p* ≤ 0.001.

**Table 5:** Chi^2^ test results on the association between gender, financial literacy, and having debt from consumer loans.

Variable	Male	Female	χ^2^	Φ
1. Consumer loans			30.93 **	0.12 ***
Yes	319 (32.0%)	223 (21.2%)		
No	679 (68.0%)	836 (78.9%)		
2. Financial literacy			24.24 **	0.11 ***
Have	244 (24.4%)	166 (15.7%)		
Do not have	754 (75.6%)	893 (84.3%)		

Note: ** *p* ≤ 0.01, *** *p* ≤ 0.001. χ^2^ = Continuity Correction. Φ = Phi-coefficient. In parentheses, the percentage of each variable’s distribution within each group.

**Table 6:** Logistic regression of having debt from consumer loans.

Variables	B	Wald	Sig.	Odds-Ratio	CI 95% L	CI 95% U
Gender	0.68	36.63	<0.001 ***	1.97	1.58	2.46
Education	−0.09	1.14	0.285	0.91	0.78	1.08
Age	0.08	21.34	<0.001 ***	1.08	1.05	1.11
Financial literacy	0.91	27.96	<0.001 ***	2.49	1.78	3.49
Anxiety	0.08	21.84	<0.001 ***	1.08	1.05	1.12
Depression	0.09	22.30	<0.001 ***	1.09	1.05	1.13
DJGLS Emotional	0.19	28.94	<0.001 ***	1.21	1.13	1.30
DJGLS Social	−0.27	56.55	<0.001 ***	0.77	0.71	0.82

Note: *** *p* ≤ 0.001. CI = confidence interval. L = lower, U = upper.

## Data Availability

The data presented in this study is available upon request from the corresponding author due to ethical restrictions. Although the data used in this study is anonymous, respondents were not informed in the information letter that their responses could be made publicly available. Therefore, for ethical reasons, the data is not publicly accessible.

## Supplementary Materials

The following supporting information can be downloaded at: https://www.mdpi.com/article/10.3390/bs16030318/s1, Supplementary file: Supplemental Analysis: Hierarchical Logistic Regression of Consumer Loan Debt.
