# Supplementary material for: No Loan Comes Without a Price: Financial Literacy, Mental Health, and Consumer Loan Use Among Young Adults in Sweden
Source: Behav Sci (Basel). 2026 Feb 26;16(3):318. doi: 10.3390/bs16030318 (PMC13024078; doi:10.3390/bs16030318)
Supplement: Supplementary file 1 [file behavsci-16-00318-s001.zip › behavsci-4141760-supplementary.pdf]

Supplemental Analysis: Hierarchical Logistic Regression of Consumer Loan Debt

A hierarchical logistic regression was conducted to examine variables associated with having consumer loans (0 = No, 1 = Yes). Model fit was assessed using Akaike Information Criterion (AIC). Variables were entered in four blocks. Block 1, containing demographic controls (gender, educational level, and age), explained 3% of the variance in the full model at that step (Nagelkerke $R^2 = 0.03$; AIC = 2338.42). Block 2 added financial literacy, increasing the total explained variance to 8% (Nagelkerke $R^2 = 0.08$; AIC = 2263.44). Block 3 added anxiety and depressive symptoms, further increasing explained variance to 16% (Nagelkerke $R^2 = 0.159$; AIC = 2149.01). Finally, Block 4 added emotional and social loneliness, bringing the total explained variance to 21% (Nagelkerke $R^2 = 0.206$; AIC = 2078.51). Each block significantly improved model fit. The results show that financial literacy, mental health, and loneliness are significantly associated with higher odds of having consumer loans, beyond demographic factors.
